# Deficiency of *miR-29b2/c* leads to accelerated aging and neuroprotection in MPTP-induced Parkinson’s disease mice

**DOI:** 10.18632/aging.203545

**Published:** 2021-09-20

**Authors:** Xiaochen Bai, Xiaoshuang Zhang, Rong Fang, Jinghui Wang, Yuanyuan Ma, Zhaolin Liu, Hongtian Dong, Qing Li, Jingyu Ge, Mei Yu, Jian Fei, Ruilin Sun, Fang Huang

**Affiliations:** 1 Department of Translational Neuroscience, Jing’an District Centre Hospital of Shanghai, State Key Laboratory of Medical Neurobiology and MOE Frontiers Center for Brain Science, Institutes of Brain Science, Fudan University, Shanghai 200032, China; 2 Department of Rehabilitation Medicine, Shanghai Jiao Tong University Affiliated Sixth People's Hospital, Shanghai 200233, China; 3 School of Life Science and Technology, Tongji University, Shanghai 200092, China; 4 Shanghai Engineering Research Center for Model Organisms, Shanghai Model Organisms Center, INC, Shanghai 201203, China

**Keywords:** Parkinson's disease, miR-29b2/c, glial cells, neuroinflammation, AMPK

## Abstract

Studies reveal a linkage of miR-29s in aging and Parkinson’s disease (PD). Here we show that the serum levels of miR-29s in 1-methyl-4-phenyl-1,2,3,6-tetrahydropyridine (MPTP)-induced PD mice exhibited dynamic changes. The role of *miR-29b2/c* in aging and PD was studied utilizing *miR-29b2/c* gene knockout mice (*miR-29b2/c* KO). *miR-29b2/c* KO mice were characterized by a markedly lighter weight, kyphosis, muscle weakness and abnormal gait, when compared with wild-type (WT) mice. The WT also developed apparent dermis thickening and adipose tissue reduction. However, deficiency of *miR-29b2/c* alleviated MPTP-induced damages of the dopaminergic system and glial activation in the nigrostriatal pathway and consequently improved the motor function of MPTP-treated KO mice. Knockout of *miR-29b2/c* inhibited the expression of inflammatory factors in 1-methyl-4-phenylpyridinium (MPP^+^)-treated primary cultures of mixed glia, primary astrocytes, or LPS-treated primary microglia. Moreover, *miR-29b2/c* deficiency enhanced the activity of AMPK but repressed the NF-κB p65 signaling in glial cells. Our results show that *miR-29b2/c* KO mice display the progeria-like phenotype. Less activated glial cells and repressed neuroinflammation might bring forth dopaminergic neuroprotection in *miR-29b2/c* KO mice. Conclusively, *miR-29b2/c* is involved in the regulation of aging and plays a detrimental role in Parkinson’s disease.

## INTRODUCTION

Parkinson’s disease (PD) is the second most frequent neurodegenerative disease, characterized by the progressive loss of dopaminergic neurons in the substantia nigra par compacta (SNpc) of the midbrain, and increased glial activation and neuroinflammation [1–3]. MicroRNAs are short noncoding RNA molecules that regulate gene expression at the post-transcriptional level [4, 5]. MicroRNAs are involved in the regulation of nervous system development, neuronal plasticity, and neurodegenerative diseases. MicroRNA29 family (miR-29s) are composed of two gene clusters: *miR-29a/b1* and *miR-29b2/c*, which are located on chromosome 6 and chromosome1, respectively, of the mouse genome. miR-29a and miR-29c only have one nucleotide difference, whereas miR-29b1 and miR-29b2 are identical in sequence [6]. miR-29s are involved in multiple biological processes. Many miR-29s target genes have been experimentally verified, including pro-cell survival genes *Bcl-2*, *Mcl-*1, *Cdc42* and *p85*-α; pro-apoptotic genes *Puma*, *Bim*, *Bak* and *Bmf*; and pro-inflammatory cytokines *IFN-γ* and *IL-12β*. In the peripheral system, studies have shown that miR-29s are involved in tissue fibrosis [7–9], metabolism and immune regulation [10–13].

miR-29s express widely in the central nervous system, and their transcripts exist both in neurons and glial cells [14, 15]. The association between miR-29s and neurological diseases has been increasingly illustrated. miR-29s are involved in the regulation of β-amyloid production, and they are down-regulated in the brain of AD patients [16, 17]; miR-29s levels also decrease in Huntington’s disease (HD) patients and HD model mice [18, 19], whereas they increase in patients with amyotrophic lateral sclerosis (ALS) and ALS mouse models [16, 20]. Both *miR-29a/b-1*-deficient mice and miR-29s down-regulated mice display an Ataxia-like phenotype [6, 21]. Regarding the effects of miR-29s expression on neuroprotection and the promotion of neuronal death in ischemic rodent models opposing results were reported [22, 23]. In previous studies, we observed that miR-29s levels were markedly decreased in the serum of PD patients with a decreasing trend related to more severe Parkinsonism [24]. Further, the miR-29s levels correlated with memory performance in PD patients [25]. We concluded that *miR-29b2/c* is linked closely to PD, but its physiological functions and pathological mechanisms involved in PD are largely unknown.

In this study, mouse serum levels of miR-29s in response to MPTP administration were measured up to 120 days post-injection. The effects of *miR-29b2/c* deficiencies on the peripheral tissues were studied utilizing *miR-29b2/c KO* mice. The dopaminergic neurotoxin MPTP was further used to induce mouse model of PD. Injuries of the nigrostriatal dopaminergic system, behavioral performance and potential mechanisms were then investigated. We observed that the miR-29s levels in the mouse serum displayed dynamic changes after MPTP administration. *miR-29b2/c* KO mice displayed accelerated aging, indicated by the lighter body weight, adipose tissue reduction, kyphosis, skin thickening, muscle weakness, and the gait abnormality. However, deficiency of *miR-29b2/c* led to the alleviated dopaminergic damage and glial activation, and consequently to the improved behavioral performance in a PD-like animal model.

## RESULTS

### The progeria-like phenotype in *miR-29b2/c* KO mice

**Figure 1:**
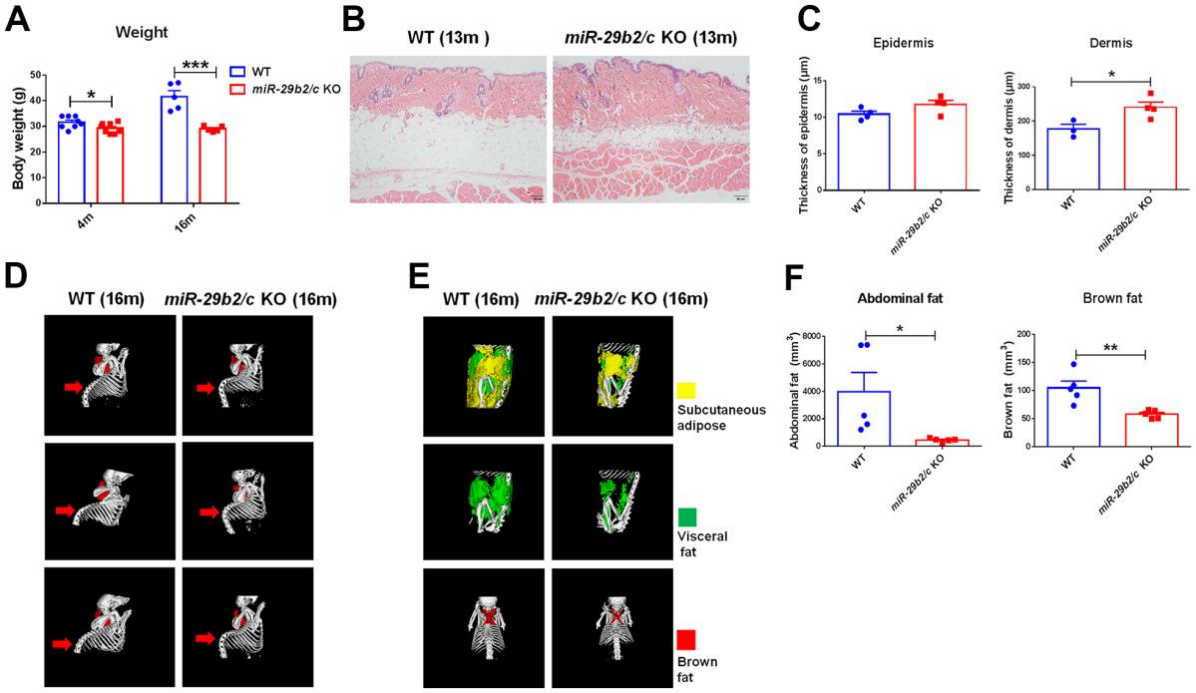
**The body weights and the peripheral characteristics of *miR-29b2/c* KO mice.** (**A**) The body weights of WT and *miR-29b2/c* KO mice at 4 and 16 months old. n=5-8. Differences were analyzed by Student-T-test. *p < 0.05 and ***p < 0.001. (**B**) H&E staining of the back skin of WT and *miR-29b2/c* KO mice at 13 months old. (**C**) The epidermis and dermis thickness of WT and *miR-29b2/c* KO mice at 13 months old. n=3-4. (**D**) microCT scan of bone of WT and *miR-29b2/c* KO mice at 16 months old. (**E**) microCT scan of abdominal fat (subcutaneous fat and visceral fat together) and brown fat of WT and *miR-29b2/c* KO mice at 16 months old. (**F**) The content and ratio analysis of abdominal and brown fat of WT and *miR-29b2/c* KO mice at 16 months old are shown. n=5. The differences were analyzed by Student-T-test. *p < 0.05, **p < 0.01.

*miR-29b2/c* knockout mice (*miR-29b2/c* KO) were generated using the CRISPR-Cas9 technique. The strategy of gene targeting and mutant mouse genotyping were presented in Supplementary Figure 1. Four- and 16-month*-old miR-29b2/c* KO mice had decreased body weights (Figure 1A). At three months of age, the features of *miR-29b2/c* KO mice remained unaltered, as proven by X-ray micro-computed tomography (microCT) scan (Supplementary Figure 2A). There were no differences in bone mineral density (BMD), trabeculae mean BMD, trabecular separation, trabecular thickness and structural model index (SMI) between *miR-29b2/c* KO mice and their WT counterpart at 13 months old (Supplementary Figure 2B). By hematoxylin and eosin (H&E) staining, we found 13-month-old *miR-29b2/c* KO mice displayed thickened dermis with increased and deepened wrinkles (Figure 1B, 1C). And kyphosis was apparent in 16-month-old *miR-29b2/c* KO mice (Figure 1C). *miR-29b2/c* KO mice at a young age (3 months old) had normal adipose tissues (Supplementary Figure 2C, 2D). However, abdominal adipose tissue (subcutaneous fat and visceral fat combined) and brown adipose tissue were dramatically reduced in *miR-29b2/c* KO mice compared to WT mice at the age of 16 months (Figure 1D). Further, the transcriptional levels of senescence markers *p21* and *p53* in the brain were analyzed. They increased markedly in the hippocampus, but not in the cortex of *miR-29b2/c* KO mice at the age of six months. p53 and p16 protein levels in the *miR-29b2/c*-deficient hippocampus did not differ from the WT controls (Supplementary Figure 3). In addition, β-galactosidase activity, a known characteristic of cellular senescence, did not differ between the brains of three-month-old WT and *miR-29b2/c* KO mice (Supplementary Figure 4).

### Muscle weakness and gait abnormality in *miR-29b2/c* KO mice

**Figure 2:**
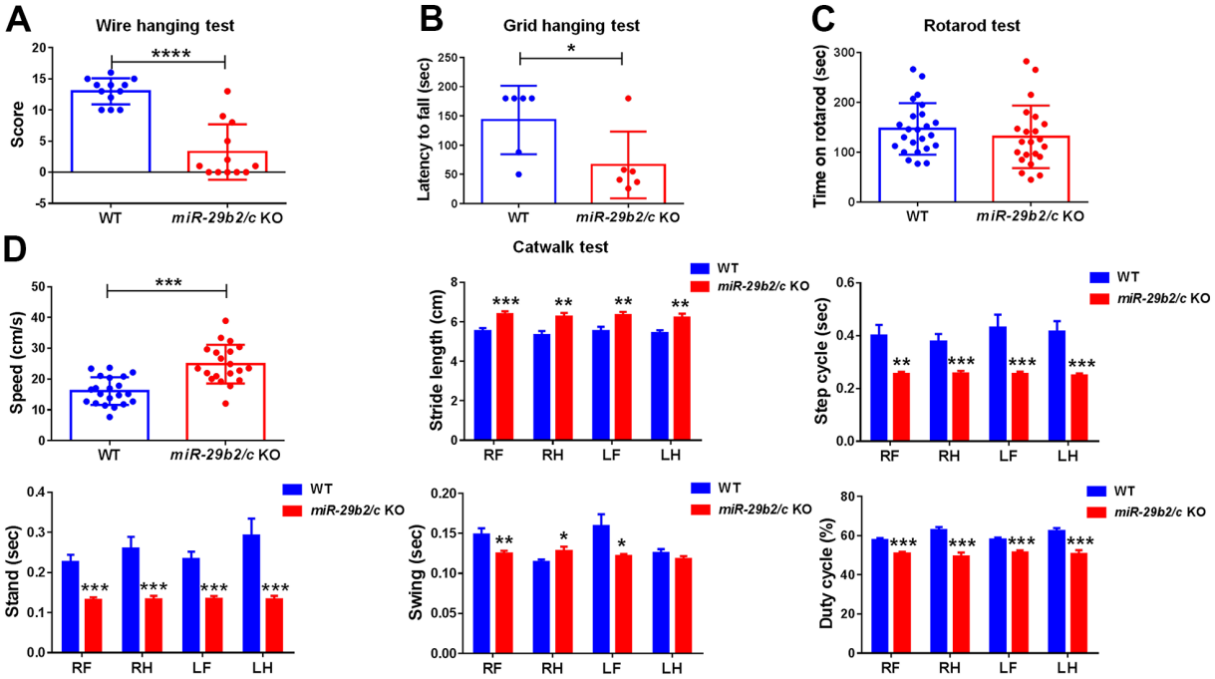
**The muscle weakness and gait abnormality in *miR-29b2/c* KO mice.** (**A**) The results of the Wire hanging test in WT and *miR-29b2/c* KO mice. n=12-14. (**B**) The results of Grid hanging test in WT and *miR-29b2/c* KO mice. n=6-13. (**C**) The results of Rotarod test in WT and *miR-29b2/c* KO mice. n=12-24. (**D**) The results of Catwalk test in WT and *miR-29b2/c* KO mice. n=12-21. The differences were analyzed by Student-T-test. *P < 0.05, **P < 0.01, ***P < 0.001 and ****P < 0.0001.

The behaviors of *miR-29b2/c*-deficient mice were assessed. Muscle strength were measured by the Wire hanging and the Grid hanging tests. The *miR-29b2/c* KO mice scored lower than the control mice in the Wire hanging test (Figure 2A). And the latency to fall was dramatically shorter in *miR-29b2/c* KO mice when compared with wild-type counterparts in the Grid hanging test (Figure 2B), but the Rotarod test performance of WT and *miR-29b2/c* KO mice did not differ significantly (Figure 2C). Animal gait was detected by Catwalk XT analysis system. Surprisingly, both speed and stride length of *miR-29b2/c* KO mice were higher than those of their counterparts, whereas the step cycle, stand and swing time were shorter, and the duty cycle reduced, in *miR-29 b2/c*-deficient mice (Figure 2D).

### Changes of miR-29s in the MPTP-induced PD mouse serum

miR-29s levels decreased in the serum of patients with PD compared to healthy controls [24]. In this study, miR-29s levels in the serum of PD mice were quantified at 3, 30 and 120 days after administration of a subacute regimen of MPTP. miR-29a and miR-29b levels decreased at 3 days after injection and recovered to baseline values at 30 days, and decreased again at 120 days. However, miR-29c level did not change significantly (Supplementary Figure 5).

### *miR-29b2/c* deficiency mitigates MPTP-induced nigrostriatal injuries and motor deficits in mice

**Figure 3:**
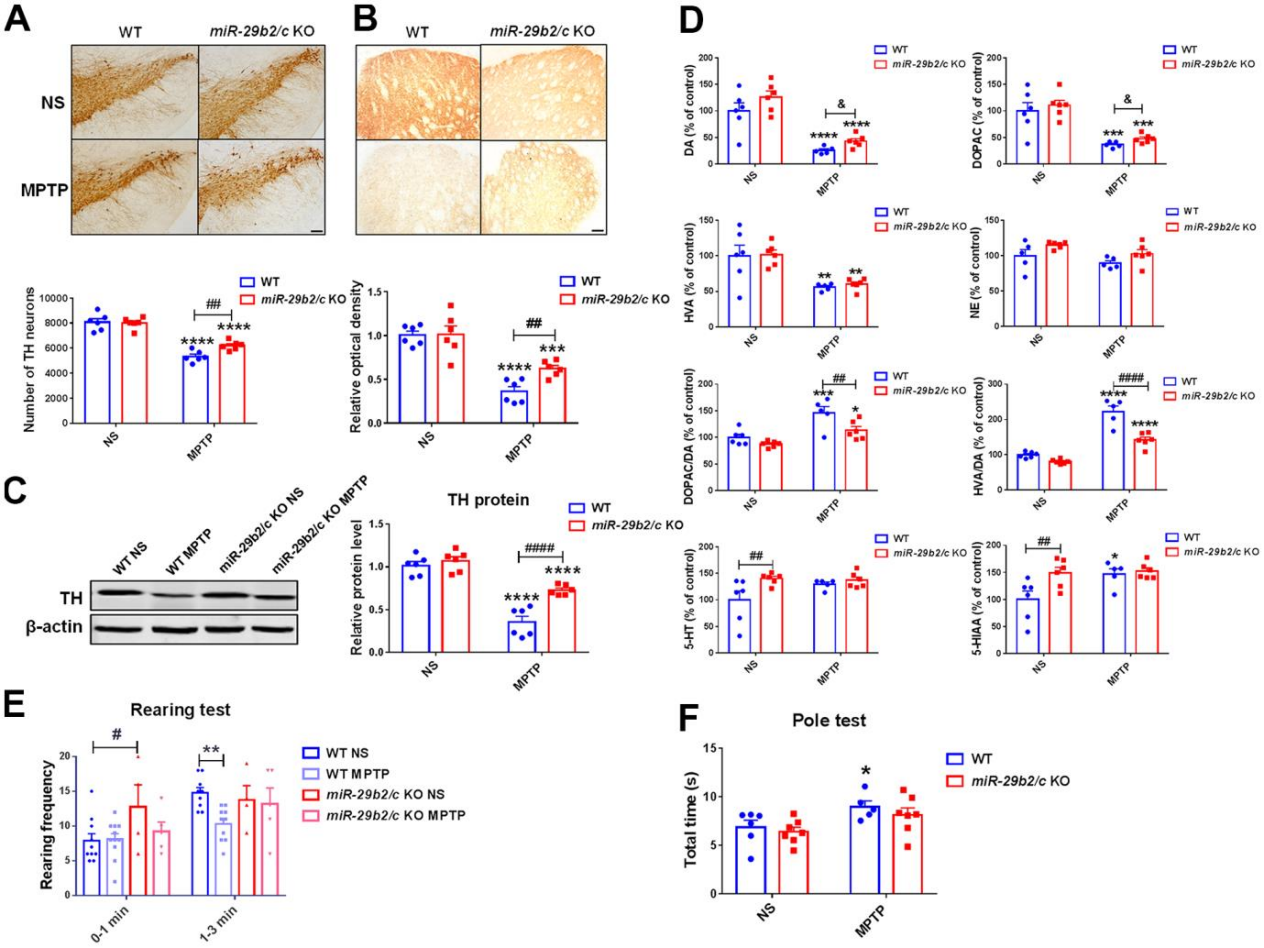
**The analysis of the nigrostriatal pathway and behavioral performance of WT and *miR-29b2/c* KO mice at 3 days after MPTP administration.** (**A**) Immunohistochemical staining of TH in the SNpc of WT and *miR-29b2/c* KO mice. Scale bar: 0.1mm. Stereological counting of TH positive dopaminergic neurons is shown in the lower panel. n=5-6. (**B**) Immunohistochemical staining showing striatal TH positive nerve fibers of WT and *miR-29b2/c* KO mice. Scale bar: 0.05mm. Densitometric analysis of the relative optical density of the staining is shown in the lower panel. n=6. (**C**) Western blot showing TH protein levels in the striatum of WT and *miR-29b2/c* KO mice. β-actin served as a loading control. The quantification of the relative TH protein levels is shown in the right panel. n=6. (**D**) Levels of striatal dopamine (DA), 5-HT, their metabolites, and norepinephrine (NE) in WT and *miR-29b2/c* KO mice. n=5-6. (**E**) The rearing frequency of WT and *miR-29b2/c* KO mice between 0-1min and 1-3min in the Rearing test. n=4-11. (**F**) The total time of WT and *miR-29b2/c* KO mice in the Pole test. n=5-7. The differences were analyzed by two-way ANOVA followed by LSD multiple comparison tests. *p<0.05, **p < 0.01, ***p<0.001 and ****p<0.0001, *vs* normal saline control. # *p* < 0.05, ## *p* < 0.01 and ####*p*<0.0001, *vs* WT group. & *p* < 0.05, by Student-T-test.

*miR-29b2/c* knockout mice and the WT littermates were injected with MPTP to induce PD model. The expression levels of *p21*, *p53* and *Pai1* in the striatum of both *miR-29b2/c* KO mice and WT controls did not change after MPTP administration (Supplementary Figure 6). MPTP exposure caused significant reductions of the TH-positive dopaminergic neurons in the SNpc [F(1, 20) = 5.441, P=0.0302], and the TH-positive nerve terminals, TH proteins [F(1, 20) = 10.5, P=0.0041] and the levels of dopamine, DOPAC and HVA in the striatum (Figure 3A–3D). However, the nigrostriatal injuries in MPTP-treated *miR-29b2/c* KO mice were dramatically alleviated when compared with MPTP-treated WT controls as the numbers of dopaminergic neurons, the densities of dopaminergic nerve terminals, and the striatal TH protein levels and dopamine concentration were significantly higher; In addition, changes in the ratios of DOPAC to DA and HVA to DA [F(1, 19) = 12.64, P=0.0021] were markedly mitigated (Figure 3A–3D). In normal saline-injected *miR-29b2/c* KO mice, the striatal concentrations of 5-HT and its metabolite 5-HIAA increased compared to their WT counterparts, and NE concentrations were close between the wild-type and *miR-29b2/c* KO mice (Figure 3D). The Rearing and the Pole tests were used to evaluate spontaneous vertical activity [26, 27] and locomotor activity of mice [28], respectively. Two days after the last MPTP injection, rearing frequency decreased in WT mice during the last 2 min when compared with normal saline-treated WT controls. Similar experiments did not reveal an effect of *miR-29b2/c* deficiency on MPTP-induced changes of rearing frequency (Figure 3E). Similarly, the total time spent on turning and climbing down increased in WT mice after MPTP administration in the Pole test, whereas the total time was close between normal saline- and MPTP-treated *miR-29b2/c* KO mice (Figure 3F).

### *miR-29b2/c* deficiency attenuates MPTP-induced glial activation in mice

**Figure 4:**
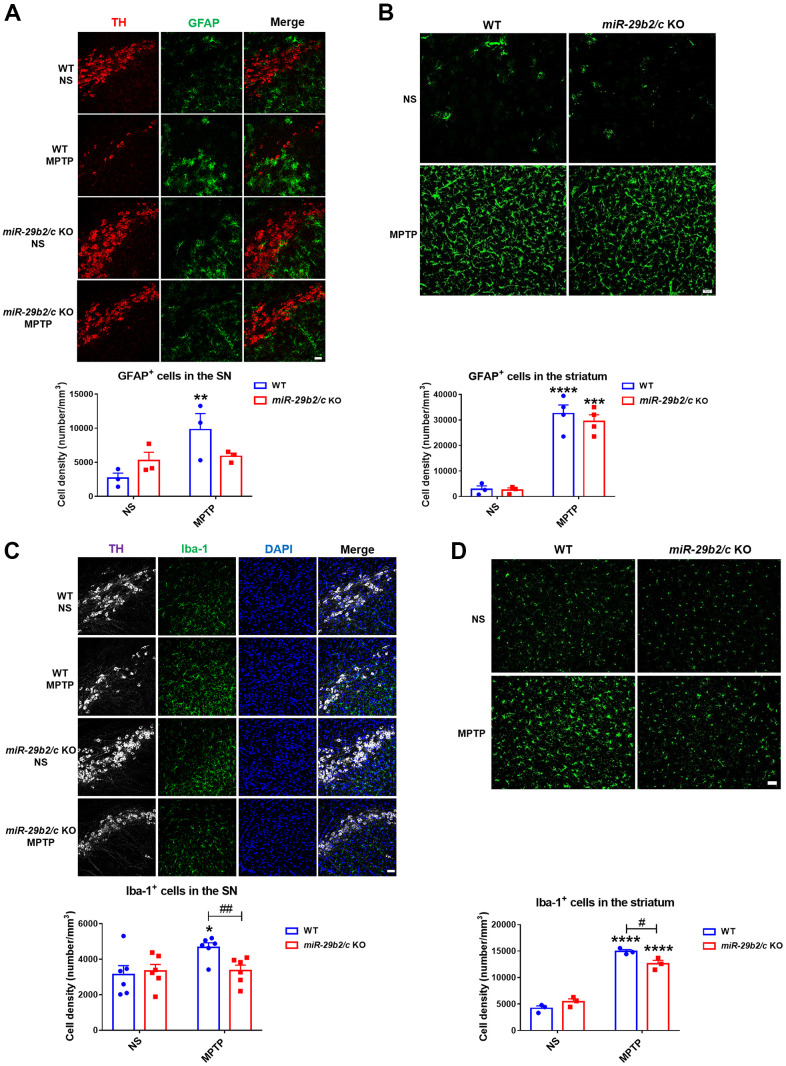
**The analysis of glial activation in the nigrostriatal pathway at 3 days after MPTP administration.** (**A**) Immunofluorescence staining for TH (red) and GFAP (green) in the SNpc of WT and *miR-29b2/c* KO mice. Scale bar: 0.1 mm. n =3-6. (**B**) Immunofluorescence staining for GFAP (green) in the striatum of WT and *miR-29b2/c* KO mice. Scale bar: 0.02mm. n =3-4. (**C**) Immunofluorescence staining for TH (white) and Iba-1 (green) in the SNpc of WT and *miR-29b2/c* KO mice. Nuclei were counterstained with DAPI (blue). Scale bar: 0.1 mm. n=6. (**D**) Immunofluorescence staining for Iba-1(green) in the striatum of WT and *miR-29b2/c* KO mice. Scale bar: 0.02mm. n=3-4. The counting of GFAP positive cells and Iba-1 positive cells in the SNpc and the striatum is shown in the lower panels. The differences were analyzed by two-way ANOVA followed by LSD multiple comparison tests. *p<0.05, **p<0.01, ***p<0.001 and ****p<0.0001, *vs* normal saline control. #*p*<0.05, *vs* WT group.

Glial cell activation and glial cell-mediated neuroinflammation are involved in PD pathology [29]. Astrocytes increased markedly in the nigrostriatal pathway of WT mice, whereas GFAP^+^ astrocytes increased in the striatum but not in the SNpc [F(1, 8) = 5.412, P=0.0484] of *miR-29b2/c* KO mice three days after MPTP injection. Notably, astrocytic densities did not differ in the two regions of WT and *miR-29b2/c* KO mice treated with MPTP (Figure 4A, 4B). Iba 1^+^ microglia increased in the substantia nigra of WT mice, and in the striatum [F(1, 8) = 12.74, P=0.0073] of both WT and *miR-29b2/c* KO mice after MPTP administration. Moreover, in *miR-29b2/c* KO mice MPTP-injection reduced microglial densities significantly (Figure 4C, 4D).

### The effects of *miR-29b2/c* deficiency in primary cultured mixed glial cells

To gain insight into the mechanisms responsible for the different outcomes of the WT and *miR-29b2/c* KO mice after MPTP injection. The effects of *miR-29b2/c* deficiency on the characteristics of glial cells were studied using a primary culture system both at baseline and after toxin exposure. The transcripts of *BDNF*, *GDNF* and *TGF-β1* increased in MPP^+^-treated WT and *miR-29b2/c* KO mixed glia (Supplementary Figure 7A, 7B). 36 h after MPP^+^ exposure, the *GDNF* expression level in *miR-29b2/c* KO mixed glia culture was significantly higher compared to WT mixed glia (Supplementary Figure 7A). MPP^+^ treatment upregulated the expression of *IL-1β*, *IL-6* and *COX-2* in mixed glia of WT mice. The treatment also increased *IL-6* expression but did not affect the transcripts of *IL-1β* and *COX-2* in *miR-29b2/c* KO mixed glia. The level of *IL-1β* in *miR-29b2/c* KO mixed glia was also lower compared to WT mixed glia 36 h after the treatment (Supplementary Figure 7C). Moreover, phosphorylated-AMPK (p-AMPK) proteins were indistinguishable between WT and *miR-29b2/c* KO mixed glia at baseline; whereas, p-AMPK levels were downregulated only in WT glia after 24 h- and 36 h-treatment of MPP^+^ (*p*=0.057 and *p*=0.09, respectively) (Supplementary Figure 7D).

### The effects of *miR-29b2/c* deficiency in primary cultured astrocytes

A scratch assay was utilized to test if deficiency of *miR-29b2/c* affected astrocytic proliferation and migration. Primary *miR-29b2/c* KO astrocytes showed no difference in the ability of proliferation and migration (Supplementary Figure 8A). The cell viability of primary WT and *miR-29b2/c* KO astrocytes manifested no difference at baseline. After MPP^+^ intoxication, the cell viability of WT astrocytes was higher compared to the controls, whereas the viability of mutant astrocytes did not change [F(1, 18) = 5.727, P=0.0278] (Supplementary Figure 8B). Enhanced ROS product and glucose uptake were observed in MPP^+^-treated WT and *miR-29b2/c* KO astrocytes (Supplementary Figure 8C, 8D).

**Figure 5:**
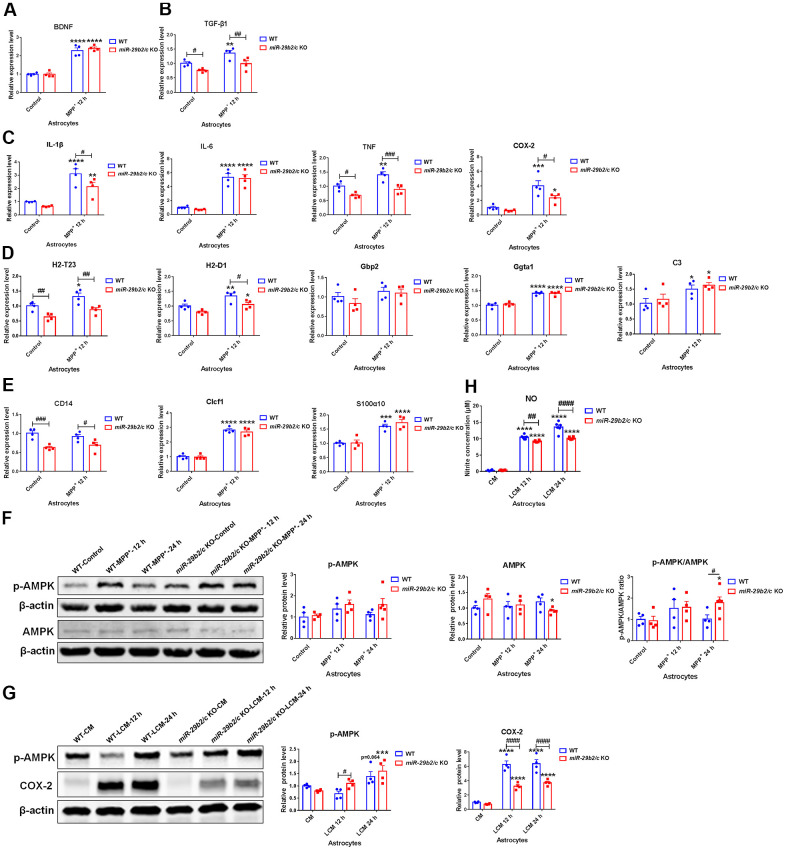
**The effects of *miR-29b2/c* deficiency in MPP^+^- and conditioned medium-treated primary astrocytes.** (**A**–**E**) qPCR analysis of *BDNF* (**A**), *TGF-β1* (**B**), *IL-1β, IL-6, TNF* and *COX2* (**C**), *H2-T23, H2-D1, Gbp2, Gpta1* and *C3* (**D**), *CD14, Clcf1* and *S100α10* (**E**) transcripts in WT and *miR-29b2/c* KO primary astrocytes treated with PBS or MPP^+^ for 12 h. n=4. (**F**) Western blot analysis of p-AMPK and AMPK protein expression in WT and *miR-29b2/c* KO primary astrocytes treated with PBS or MPP^+^ for 12 h and 24 h. β-actin served as a loading control. Quantifications of relative p-AMPK and AMPK protein levels and their ratio are shown in the right panel. n=4-5. (**G**) Western blot analysis of p-AMPK and COX-2 protein expression in WT and *miR-29b2/c* KO primary astrocytes exposed to conditioned medium (CM) or LPS-treated conditioned medium (LCM) of BV2 cells for 12 h and 24 h. β-actin served as a loading control. Quantifications of relative p-AMPK and COX-2 protein levels are shown in the right panel. n=3-4. (**H**) Nitrite concentration in the culture medium of WT and *miR-29b2/c* KO primary astrocytes treated with CM or LCM of BV2 cells for 12 h and 24 h. The differences were analyzed by two-way ANOVA followed by LSD multiple comparison tests. *p<0.05, **p < 0.01, ***p<0.001 and ****p<0.0001, *vs* PBS control. # *p* < 0.05, ## *p* < 0.01, ### *p*<0.001 and #### *p*<0.0001, *vs* WT group.

The expression levels of neurotrophic factors, inflammation-related molecules, astrocytic A1 type and A2 type marker genes in MPP^+^-treated astrocytes were measured. At 12 h after the challenge, the expression of *BDNF* was elevated in the WT and *miR-29b2/c* KO astrocytes (Figure 5A). *TGF-β1* expression level was significantly upregulated in WT astrocytes after the exposure to MPP^+^, while it was downregulated in *miR-29b2/c* KO astrocytes when compared with wild-type astrocytes both at baseline and after the intoxication (Figure 5B). The base level of *TNF* transcript was lower in *miR-29b2/c* KO astrocytes when compared with WT astrocytes. After 12 h-treatment, the expression levels of *IL-1β*, *IL-6* and *COX-2* increased significantly in WT and *miR-29b2/c* astrocytes, whereas *TNF* transcript was elevated only in WT astrocytes, and *IL-1β*, *TNF*, and *COX-2* transcripts decreased in *miR-29b2/c* KO astrocytic cells when compared with wild-type counterparts (Figure 5C). A1 marker *H2-T23* and A2 marker *CD14* were markedly lower in PBS-treated *miR-29b2/c* KO astrocytes when compared with WT controls. After 12 h-treatment, A1 markers *H2-D1*, *Ggta1* and *C3*, and A2 markers *Clcf1* and *S100α10* were upregulated both in WT and *miR-29b2/c* KO astrocytes; *H2-T23* increased only in WT astrocytes; *Gbp2* and *CD14* did not change in the two genotypes of astrocytes. Moreover, transcripts of *H2-T23*, *H2-D1* and *CD14* were downregulated in *miR-29b2/c* KO astrocytes when compared with WT controls (Figure 5D, 5E). 12 h and 24 h after the treatment, the levels of AMPK proteins and phosphorylated-AMPK proteins did not alter in WT astrocytes, whereas, at 24 h after MPP^+^ challenge, the AMPK protein level decreased in *miR-29b2/c* KO astrocytes, and the ratio of p-AMPK to AMPK in mutant astrocytes increased in comparison with WT astrocytes and PBS-treated mutant astrocytes as well (Figure 5F). Additionally, the conditioned medium from BV2 cells treated with PBS or LPS (shortly named as CM and LCM) was used to stimulate primary astrocytes of WT and *miR-29b2/c* KO mice. Though the expression of p-AMPK protein was not changed after 12 h-treatment of LCM, it was significantly higher in *miR-29b2/c* KO astrocytes compared with WT astrocytes. We observed that the p-AMPK proteins increased in both genotypes of astrocytes after 24 h-treatment of LCM. LCM stimulation markedly increased the expression of COX-2 proteins [F(2, 18) = 10.29, P=0.0010], however, the COX-2 protein level in *miR-29b2/c* KO astrocytes decreased compared with WT astrocytes (Figure 5G). Nitrite concentration in LCM-treated *miR-29b2/c* KO astrocytes was also significantly lower compared with LCM-treated WT astrocytes [F(2, 30) = 17, P<0.0001] (Figure 5H). Further, the expression of senescence marker genes was evaluated. 12 h-treatment of MPP^+^ increased *p53* transcript levels in both WT and *miR-29b2/c* KO astrocytes. *p19* and *Pai1* transcripts were elevated only in WT astrocytes, and *Pai1* expression levels in *miR-29b2/c* KO astrocytes were decreased compared to WT astrocytes after the treatment (Supplementary Figure 9A). Moreover, Bcl-2, Bax protein levels and the ratio of Bcl-2 to Bax did not change in WT and *miR-29b2/c* KO astrocytes (Supplementary Figure 9B).

### The effects of *miR-29b2/c* deficiency in primary cultured microglia

**Figure 6:**
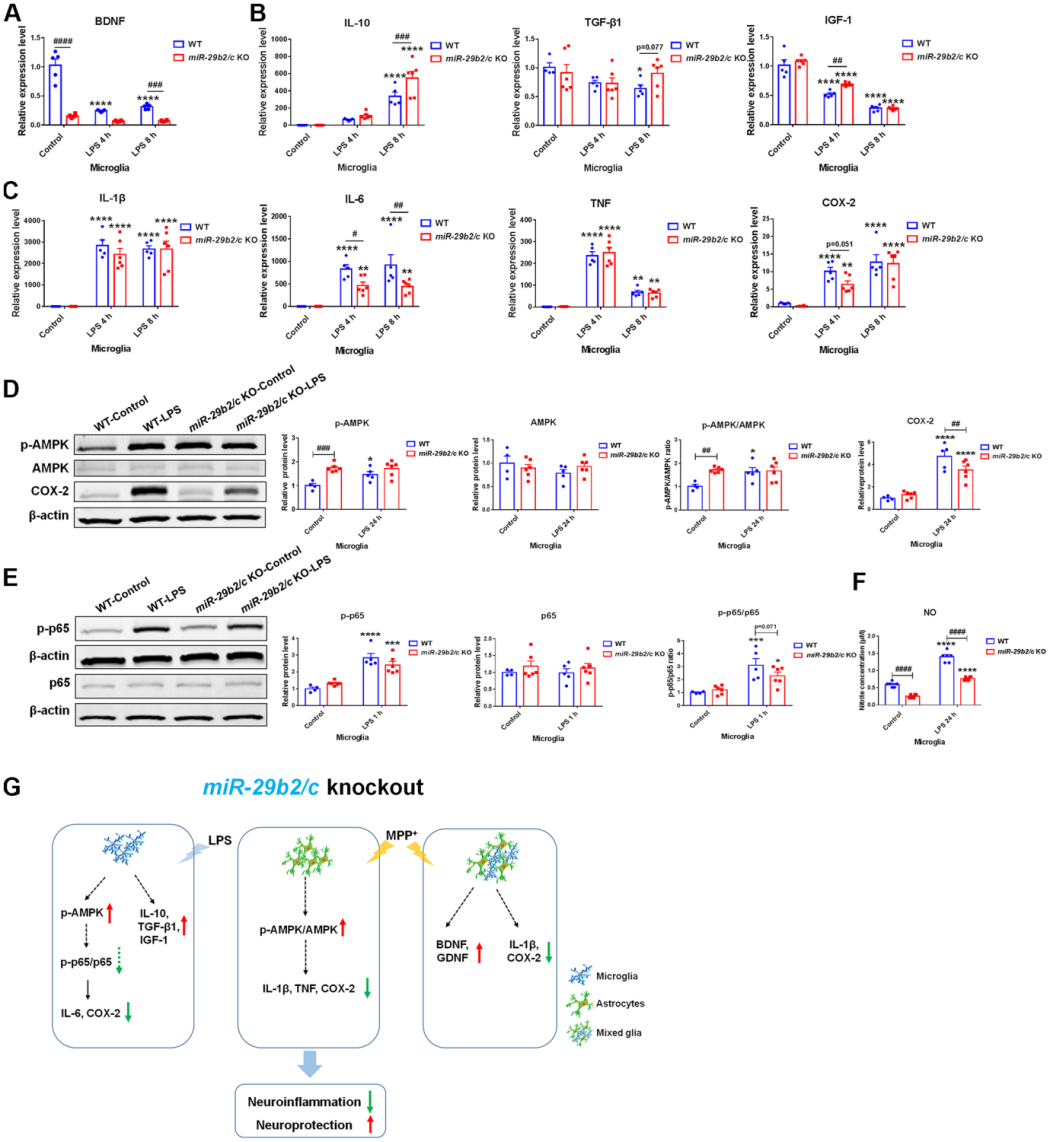
**The effects of *miR-29b2/c* deficiency in LPS-treated primary microglia.** (**A**) qPCR analysis of *BDNF* (A), *IL-10 TGF-β1* and *IGF-1* (**B**), and pro-inflammatory factors *IL-1β*, *IL-6*, *TNF* and *COX2* (**C**) in WT and *miR-29b2/c* KO primary microglia treated with PBS or LPS for four and eight hours. n=4-6. (**D**) Western blot analysis of p-AMPK, AMPK and COX-2 protein expression in WT and *miR-29b2/c* KO primary microglia treated with PBS or LPS for 24 h. β-actin served as a loading control. Quantifications of relative p-AMPK, AMPK and COX-2 protein level and the ratio of p-AMPK to AMPK are shown in the right panel. n=4-6. (**E**) Western blot analysis of p-p65 and p65 protein expression in WT and *miR-29b2/c* KO primary microglia treated with PBS or LPS for one hour. Quantifications of relative p-p65 and p65 protein level and their ratio are shown in the right panel. n=4-6. (**F**) Nitrite concentration in the culture medium of WT and *miR-29b2/c* KO microglia treated with PBS or LPS for 24 h. n=6. The differences were analyzed by two-way ANOVA followed by LSD multiple comparison tests. *p<0.05, **p < 0.01, ***p<0.001 and ****p<0.0001, *vs* PBS control. # *p* < 0.05, ## *p* < 0.01, ### *p*<0.001 and ####*p*<0.0001, *vs* WT group. (**G**) Diagram of effects of *miR-29b2/c* deficiency in Parkinson’s disease.

To measure the response of *miR-29b2/c-*deficient microglia to inflammatory stimuli, LPS was used. *BDNF* expression level was lower in *miR-29b2/c* KO microglia at baseline. Four and eight hours after LPS treatment, the amount of *IL-1β*, *IL-6*, *TNF*, and *COX-2* transcripts were upregulated, while *IGF-1* expression was downregulated in WT and *miR-29b2/c* KO microglial cells, the expression of *BDNF* only decreased in WT microglia [F(2, 27) = 41.5, P<0.0001]. *IL-10* transcript increased in WT and *miR-29b2/c* KO microglia [F(2, 27) = 3.753, P=0.0365], and *TGF-β1* transcript decreased in WT microglia at eight hours after the LPS challenge. *BDNF* and *TGF-β1* transcripts were not changed in *miR-29b2/c* KO microglia. In addition, *IL-6* transcripts were significantly reduced in *miR-29b2/c* KO microglia after the challenge compared to WT controls, *IGF-1* and *IL-10* transcripts were markedly higher in *miR-29b2/c* KO microglia after four and eight hours of intoxication, respectively (Figure 6A–6C). At baseline, the p-AMPK protein level and p-AMPK to AMPK ratio were dramatically elevated in *miR-29b2/c* KO microglia when compared with WT microglia. 24 h after the treatment of LPS, p-AMPK level [F(1, 17) = 5.066, P=0.0379] and the ratio [F(1, 17) = 5.537, P=0.0309] were elevated only in WT microglia (Figure 6D). At one hour after the treatment, the phosphorylated-NF-κB p65 (p-p65) proteins and p-p65 to p65 ratio, but not p65 proteins, increased in wild-type and *miR-29b2/c* KO microglial cells, yet the ratio decreased modestly in *miR-29b2/c* KO microglial cells when compared with WT microglia (*p*=0.071) (Figure 6E). The expression of COX-2 was enhanced in the two genotypes of microglial cells; whereas, the level of COX-2 in mutant microglia decreased when compared with WT controls after 24 h-treatment of LPS [F(1, 17) = 5.33, P=0.0338] (Figure 6D). Nitrite product was induced by the treatment of LPS in both genotypes of microglia, however, it was dramatically lower in *miR-29b2/c* KO microglia at baseline and after the treatment of LPS [F(1, 20) = 14.03, P=0.0013] (Figure 6F).

## DISCUSSION

The expression of miR-29 family members is upregulated in multiple tissues when individuals are getting older [30–32]. Works from our lab and others have shown a linkage between miR-29s and Parkinson’s disease [33]. In the present study, the roles of *miR-29b2/c* in aging and Parkinson’s disease were studied. miR-29s exert pro- and anti-aging effects depended on tissues, species and stages of development. We found that 3-month-old *miR-29b2/c* KO mice had a normal skeleton and adipose tissue, and 4-month-old *miR-29b2/c* KO mice displayed lighter body weight compared with their WT counterpart, which is in agreement with other studies [11, 34]. Muscle weakness and unstable gait were detected in 3-month-old *miR-29b2/c* KO mice. Thirteen-month-old *miR-29b2/c* KO mice showed dermis thickening. However, they had the normal fine structure of femur bone. The reduction of body weight existed in 16-month-old *miR-29b2/c* KO mice. At this age, *miR-29b2/c* KO mice had less abdominal fat and brown fat, and exhibited apparent kyphosis. miR-29s are involved in p53-mediated cell cycle arrest [35, 36] and p16/Rb-driven cellular senescence [37]. However, cellular senescence was not evident in the *miR-29b2/c* KO mouse brain. Therefore, though *miR-29b2/c* contributes to the regulation of aging, its roles in the peripheral tissues and the brain might be different.

The serum levels of miR-29s were downregulated in patients with PD compared to healthy controls [24]. Here, we found that miR-29s expression in PD mouse serum fluctuated from 3 to 120 days after MPTP administration. miR-29s are abundant in the brain [6, 30]. Via going through GEO profile, miR-29c expression is found to be upregulated in the substantia nigra of PD patients (*p*=0.0059, by Mann-Whitney test), moreover, miR-29c expression in the superior frontal gyrus did not differ (*p*=0.47, by Student-T-test), when compared with the control subjects (Supplementary Figure 10). Here, *miR-29b2/c* KO mice were challenged with MPTP to induce PD-like injuries. The mutant mice showed less severe injuries to the nigrostriatal dopaminergic system and milder glial activation, and subsequently behavioral resistance to some extent. Thus, *miR-29b2/c* has a detrimental role in the pathology of PD. We observed that in the MPTP-induced mouse PD model, the striatal levels of *p21*, *p53* and *Pai1* transcripts did not change, suggesting cellular senescence might not occur. Aging is regarded as a high risk factor for the development of PD [38], however, aging-related changes in the brain, especially the dopaminergic system might be a more relevant factor.

Primary glia from WT and *miR-29b2/c* KO mice were cultured to investigate the underlying mechanisms. MPP^+^ treatment increased *GDNF* expression, and decreased *IL-1β* expression in the *miR-29b2/c*-deficient mixed glia. MPP^+^ also increased the expression of pro-inflammatory genes in WT astrocytes but not in *miR-29b2/c*-deficient astrocytes. Likewise, mutant astrocytes produced less NO compared with WT astrocytes after being exposed to the conditioned medium of BV2 cells treated with LPS. In primary microglia culture, compared to LPS-treated WT controls, the transcripts of anti-inflammation cytokine *IL-10* and neurotrophic factor *IGF-1* were markedly higher, and the transcripts of pro-inflammation cytokine *IL-6* decreased in LPS-treated *miR-29b2/c* KO microglia. NO levels were decreased in *miR-29b2/c* KO microglia at baseline and after the intoxication of LPS. Studies have shown that miR-29s and many predicted target genes are involved in metabolic processes [34, 39–41]. As an essential regulator, AMPK has been proven to be protective in PD when activated [42]. Activation of AMPK stimulates Sirtuin 1, and inhibits NF-κB activation and downstream inflammatory target genes indirectly [43]. We observed that the phosphorylated-AMPK protein level and the ratio of p-AMPK to AMPK were upregulated in LCM-treated and MPP^+^-treated *miR-29b2/c* KO astrocytes respectively. The COX-2 protein level was decreased in LCM-treated mutant astrocytes compared to WT astrocytes. At baseline, AMPK activity was elevated in *miR-29b2/c* KO microglia. The amount of COX-2 protein was significantly reduced in *miR-29b2/c* KO microglia when compared with WT controls after the treatment of LPS. Under LPS treatment, the p-p65 and p65 ratio was slightly downregulated in the mutant microglia, implied a mitigated NF-κB signaling pathway. Our results indicate that enhanced AMPK activity and reduced inflammatory response in glia protect the nigrostriatal pathway in *miR-29b2/c* KO mice (Figure 6G). Conclusively, *miR-29b2/c* plays important roles in aging and damage in the nigrostriatal dopaminergic system.

## MATERIALS AND METHODS

### Mice

*miR-29b2/c* KO mice and WT littermates were obtained from Shanghai Research Center for Model Organisms, China. Experimental protocols were approved by the Institutional Animal Care and Use Committee of Fudan University. Surgeries were conducted under general anesthesia. All efforts were taken to reduce adverse effects.

Mice were administered intraperitoneally with MPTP-HCl (Sigma, USA) at 20 mg/kg or normal saline (NS) for 5 consecutive days as described [44].

### RNA and miRNA extraction, and quantitative PCR (qPCR)

The methods of RNA and miRNA extraction, reverse transcription, and qPCR were referred to our previous work [24, 45]. The primers for qPCR were listed in Table 1.

**Table 1:** Primers for qPCR analysis.

**Name**	**Sequence (5’ 3’)**
Mouse actin F	CAGGATGCAGAAGGAGATTAC
Mouse actin R	AACGCAGCTCAGTAACAGTC
Mouse BDNF F	TCATACTTCGGTTGCATGAAGG
Mouse BDNF R	AGACCTCTCGAACCTGCCC
Mouse CD14 F	GGACTGATCTCAGCCCTCTG
Mouse CD14 R	GCTTCAGCCCAGTGAAAGAC
Mouse Clcf1 F	CTTCAATCCTCCTCGACTGG
Mouse Clcf1 R	TACGTCGGAGTTCAGCTGTG
Mouse COX2 F	GTTCATCCCTGACCCCCAAG
Mouse COX2 R	ACTCTGTTGTGCTCCCGAAG
Mouse GDNF F	GACGTCATGGATTTTATTCAAGCCACC
Mouse GDNF R	CTGGCCTACTTTGTCACTTGTTAGCCT
Mouse Gbp2 F	GGGGTCACTGTCTGACCACT
Mouse Gbp2 R	GGGAAACCTGGGATGAGATT
Mouse Ggta1 F	GTGAACAGCATGAGGGGTTT
Mouse Ggta1 R	GTTTTGTTGCCTCTGGGTGT
Mouse H2-D1 F	TCCGAGATTGTAAAGCGTGAAGA
Mouse H2-D1 R	ACAGGGCAGTGCAGGGATAG
Mouse H2-T23 F	GGACCGCGAATGACATAGC
Mouse H2-T23 R	GCACCTCAGGGTGACTTCAT
Mouse IGF-1 F	AGAGCCTGCGCAATGGAATAAAGT
Mouse IGF-1 R	TTGGTGGGCAGGGATAATGAGG
Mouse IL-1β F	GCAACTGTTCCTGAACTC
Mouse IL-1β R	CTCGGAGCCTGTAGTGCA
Mouse IL-6 F	CATAGCTACCTGGAGTACATGA
Mouse IL-6 R	CATTCATATTGTCAGTTCTTCG
Mouse IL-10 F	AGCCGGGAAGACAATAACTG
Mouse IL-10 R	GGAGTCGGTTAGCAGTATGTTG
Mouse iNOS F	CCCTTCCGAAGTTTCTGGCAGCAGC
Mouse iNOS R	GGCTGTCAGAGCCTCGTGGCTTTGG
Mouse p21 F	GTGGGTCTGACTCCAGCCC
Mouse p21 R	CCTTCTCGTGAGACGCTTAC
Mouse p19^Arf^ F	GCCGCACCGGAATCCT
Mouse p19^Arf^ R	TTGAGCAGAAGAGCTGCTACGT
Mouse Pai1 F	TCAGAGCAACAAGTTCAACTACACTGAG
Mouse Pai1 R	CCCACTGTCAAGGCTCCATCACTTGCCCA
Mouse p53 F	GAGTATACCACCATCCACTACAAG
Mouse p53 R	GCACAAACACGAACCTCAAAG
Mouse S100α10 F	CCTCTGGCTGTGGACAAAAT
Mouse Slc10α6 R	CCACAGGCTTTTCTGGTGAT
Mouse TGF-β1 F	CCTGAGTGGCTGTCTTTTGA
Mouse TGF-β1 R	CGTGGAGTTTGTTATCTTTGCTG
Mouse TNF F	CACGCTCTTCTGTCTACTGAACTTC
Mouse TNF R	GCAGCCTTGTCCCTTGAAGAGAACC
Mouse YM1 F	GTCACAGGTCTGGCAATTC
Mouse YM1 R	GTAGAGACCATGGCACTG

### Western blot

Protein samples were separated by SDS-PAGE and transferred onto PVDF membranes (Millipore, USA). Primary antibodies were: rabbit phospho-AMPKα (Thr172) (1:1000; Cell Signaling Technology, USA), mouse anti-AMPK (1:1000; Proteintech, USA), rabbit anti-Bax (1:1000; Cell Signaling Technology, USA), rabbit anti-Bcl-2 (1:500; Cell Signal Technology, USA), rabbit anti-Sirtin1(1:1000, Millipore, USA), rabbit anti-COX2 (1:1000; Abcam, USA), rabbit anti-GFAP (1:1000; Dako, Japan), mouse anti-β-actin (1:1000; Santa Cruz Biotechnology, USA), mouse anti-NF-κB p65 (1:1000; Santa Cruz Biotechnology, USA), and rabbit anti-phospho-NFκB p65 (1:1000; Cell Signaling Technology, USA). Protein bands were detected with an Odyssey infrared imaging system (Li-Cor, USA).

### Stereological cell counting, and quantification of Iba1^+^and GFAP^+^ cells

TH^+^ neurons in the SNpc were quantified as previously described [46]. Stereological counting was performed by two operators blinded to mouse groups.

To quantify Iba1^+^and GFAP^+^ cells, cell counting with Image-Pro Plus 6.0 (Media Cybernetics, USA) was performed as described [47].

### HPLC

The striatal concentrations of monoamines (DA, 5-HT and NE) and their metabolites (DOPAC, HVA and 5-HIAA) were determined by HPLC as described [48].

### Behavioral tests

### Rotarod test

The Rotarod test was performed in reference to previous studies [48, 49].

### Pole test

The Pole test was conducted as previously described [48]. A mouse was put head-up near the top of a pole (1 cm in diameter and 80 cm in height). Both the time to turn and time to climb down were recorded.

### Wire hanging test

A mouse was suspended by its forelimbs from a 50 cm wide 2-mm thick metallic wire and subjected to a 180-sec hanging test as described [50].

### Grid hanging test

Grid hanging test was carried out as described [51]. A mouse was put on a grid. The grid was then turned upside down. Latency to fall was recorded. The trial ended after 3 min.

### Catwalk test

After habituating to the CatWalk XT gait analysis system (Noldus, Netherlands), a mouse was allowed to cross the recording field of the runway (40 cm in length) in both directions with three independent attempts. Runs for analysis were chosen based on a minimum of five-step cycles. CatWalk software was used to classify the footprints.

### Rearing test

A mouse was placed in a 400 mL glass beaker, and the rearing events were counted for a 3 min time course.

### Cultures of primary astrocyte and microglial cells

Primary astrocyte and microglia were isolated from neonatal mice at P1-P3 as described [52]. After a culture of two weeks, astrocytic cells were purified by shaking at 200 rpm for 12 h. The purification of microglia was done as described [53]. On day 21, cultures were trypsinized (0.0625% trypsin) at 37° C for 40 min. Microglia were cultured with the mixed glial supernatants.

### NO assay

The nitrite in the supernatant was detected by using Griess reagent (Beyotime, China) according to manufacturer instructions. Nitrite concentrations were calculated with reference to the standard curve generated with NaNO_2_.

### Reactive oxygen species (ROS) assay

Dihydroethidium (DHE) (Sigma, USA) was used to probe superoxide radicals. Astrocytes were plated in 96-well plates at 5x10^4^ per well, and treated with 1mM MPP^+^ for different hours. Cells were then washed with PBS and incubated with 5 μM DHE at 37° C for 25 min. After washing, the fluorescence intensity was measured at an excitation wavelength of 485 nm and an emission wavelength of 512 nm as described [54].

### Senescence-associated β-galactosidase (SA-β-gal) assay

The SA-β-gal assay was conducted using Senescence β -Galactosidase Staining Kit (Cell Signaling Technology, USA) according to manufacturer instructions.

### X-ray micro-computed tomography (microCT) scan

Three-dimensional structures of a mouse were obtained by high-resolution X-ray microCT scanning (PerkinElmer, USA). Volumes were quantified using the region of interest module (AnalyzeDirect, USA).

### Statistical analysis

Data were shown as the means ± SEM. All data were assessed for normal distribution by the Shapiro-Wilk test. When equal variances assumed, statistical significance was assessed by two-tailed Student’s T-test for two groups, or Two-way ANOVA followed by LSD multiple comparisons for three or more groups using Prism 7 software (GraphPad Software Inc., San Diego, USA). *P* < 0.05 was considered statistically significant. Corresponding values of significant interaction were presented for Figure 3–6 and Supplementary Figure 8 in the text.

## Supplementary Material

Supplementary Figures
