# Supplementary material for: Deficiency of miR-29b2/c leads to accelerated aging and neuroprotection in MPTP-induced Parkinson’s disease mice
Source: Aging (Albany NY). 2021 Sep 20;13(18):22390–411. doi: 10.18632/aging.203545 (PMC8507277; doi:10.18632/aging.203545)
Supplement: Supplementary Figures [file aging-13-203545-s001.pdf]

SUPPLEMENTARY FIGURES

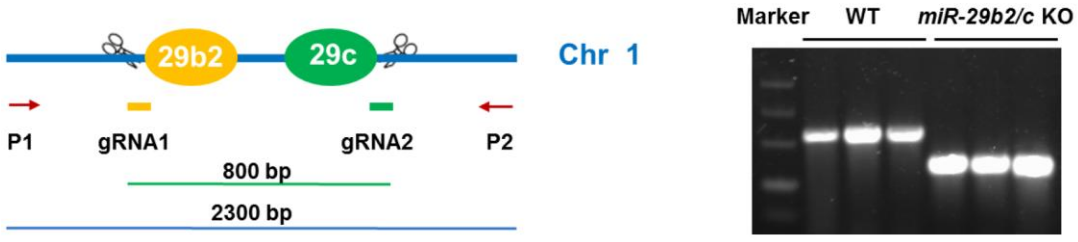

Supplementary Figure 1. The strategy of *miR-29b2/c* knockout in mice and identification of *miR-29b2/c* KO mice.

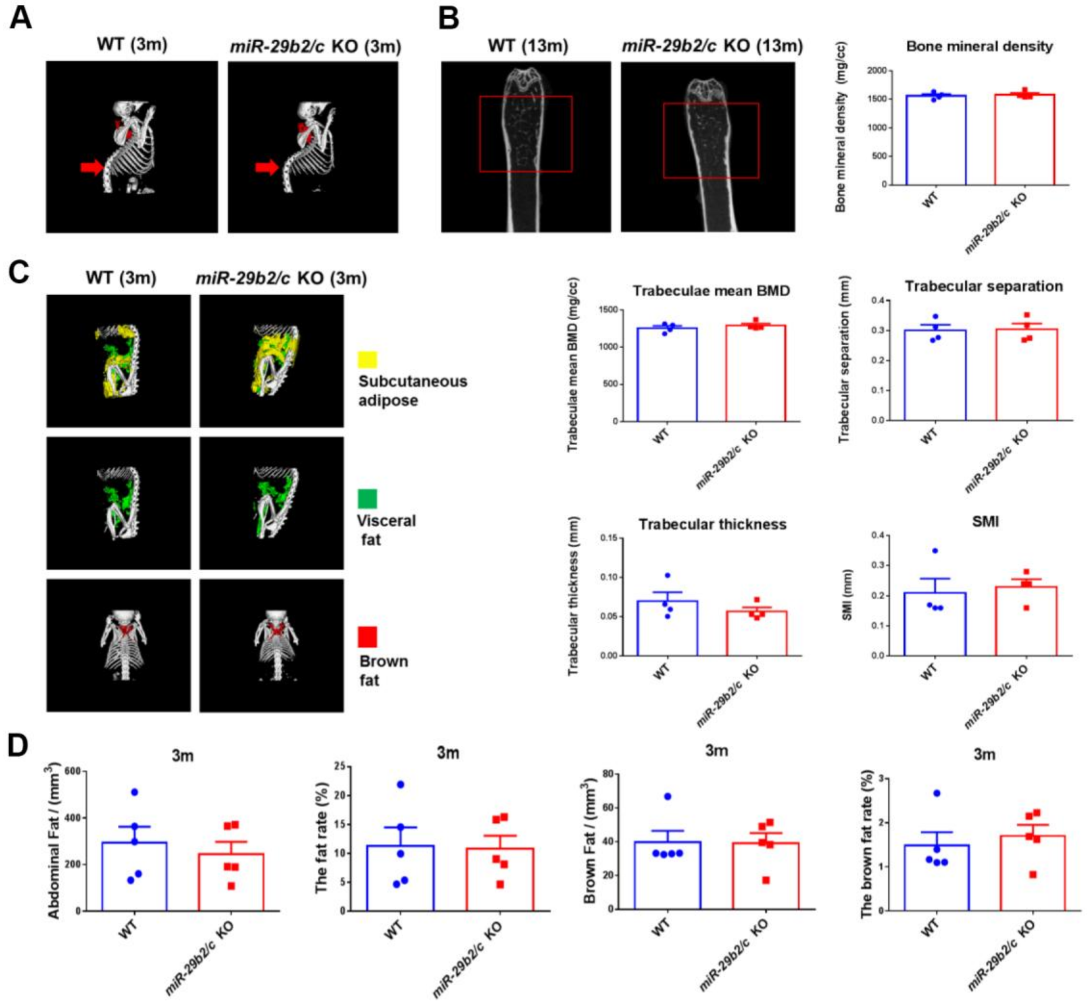

Supplementary Figure 2. Peripheral characteristics of WT and *miR-29b2/c* KO mice. (**A**) micro-CT scan of bone of WT and *miR-29b2/c* KO mice at 3 months old. (**B**) microCT scan of trabecular bone of WT and *miR-29b2/c* KO mice at 13 months old. Bone mineral density (BMD), trabecular mean BMD, trabecular separation, trabecular thickness and structural model index (SMI) are also shown. mg/cc: milligram/cubic centimeter; mm: millimeter. n=4. (**C**) microCT scan of abdominal fat (subcutaneous fat and visceral fat together) and brown fat of WT and *miR-29b2/c* KO mice at 3 months old. (**D**) The content and ratio analysis of abdominal fat and brown fat are shown. n=5.

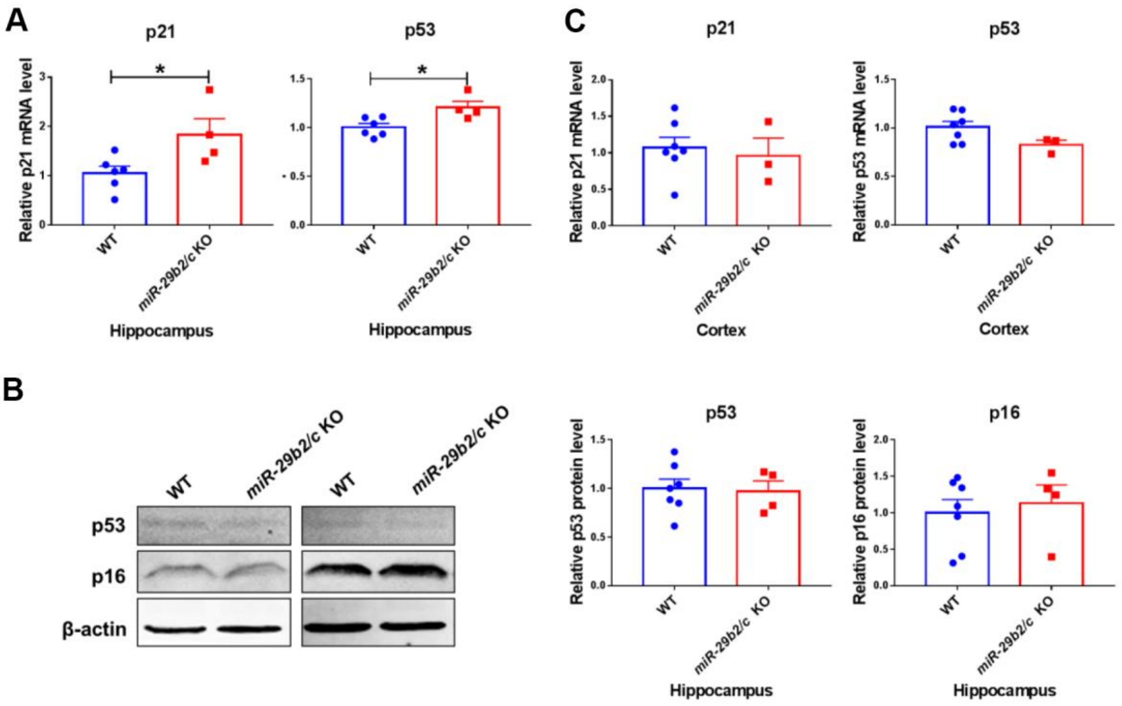

Supplementary Figure 3. The expression of senescence marker genes in the hippocampus and cortex of WT and *miR-29b2/c* KO mice at 6 months old. (**A**) qPCR analysis of *p21* and *p53* transcripts in the hippocampus. The differences were analyzed by Student-T-test. n=4-6. **p* < 0.05. (**B**) Western blot analysis of p53 and p16 protein expression in the hippocampus. β-actin served as a loading control. Quantification of relative p53 and p16 expression levels are shown in the right panel. n=4-7. (**C**) qPCR analysis of *p21* and *p53* transcripts in the cortex. n=3-7.

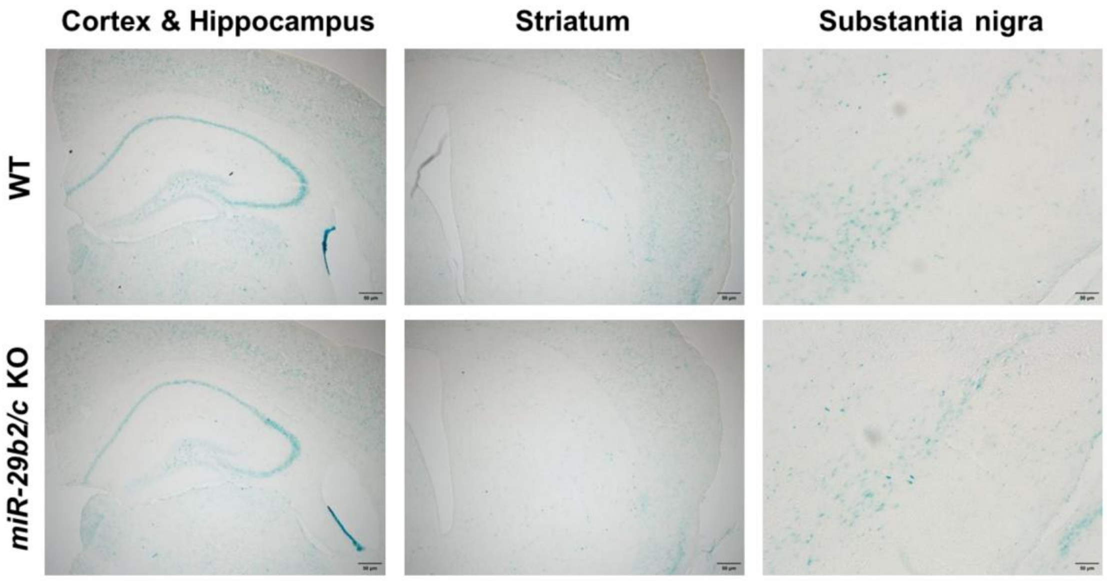

Supplementary Figure 4. β-galactosidase activity in the cortex, hippocampus, striatum and substantia nigra of 3-month-old WT and *miR-29b2/c* KO mice. Scale bar: 50 μm.

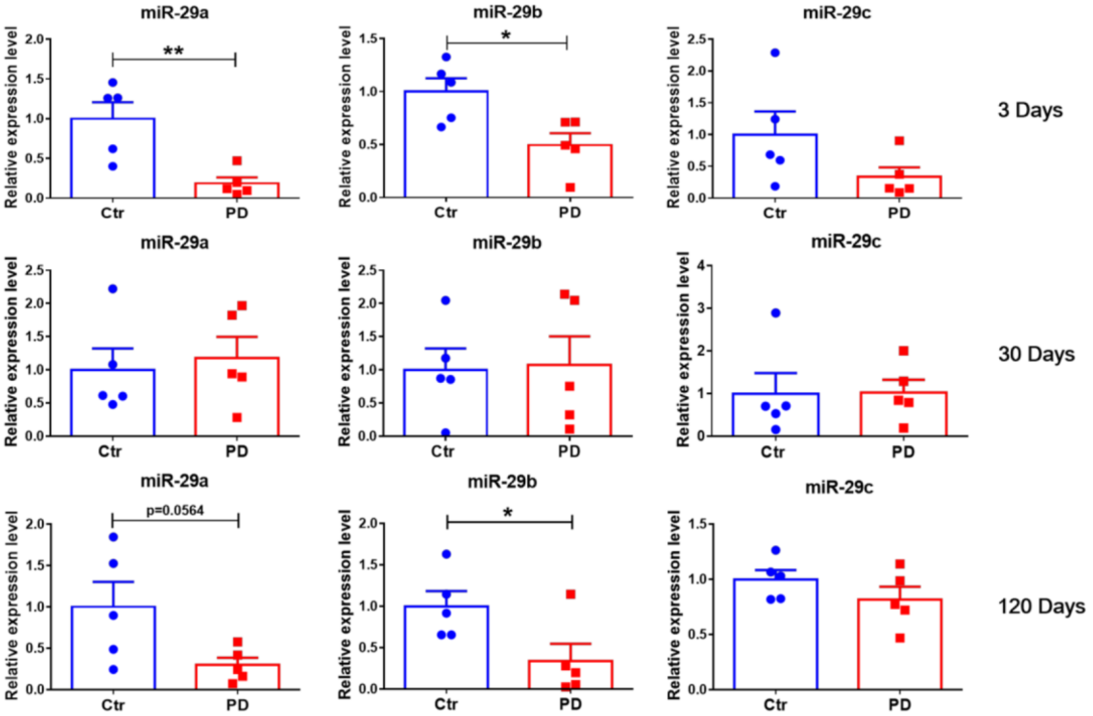

Supplementary Figure 5. The expression levels of miR-29s in the serum of MPTP-induced PD mice. miR-29s levels in the serum of control and PD mice at 3, 30 and 120 days after the administration of subacute regimen of MPTP were shown. n=5. The differences were analyzed by Student-T-test. *$p< 0.05$ and **$p< 0.01$.

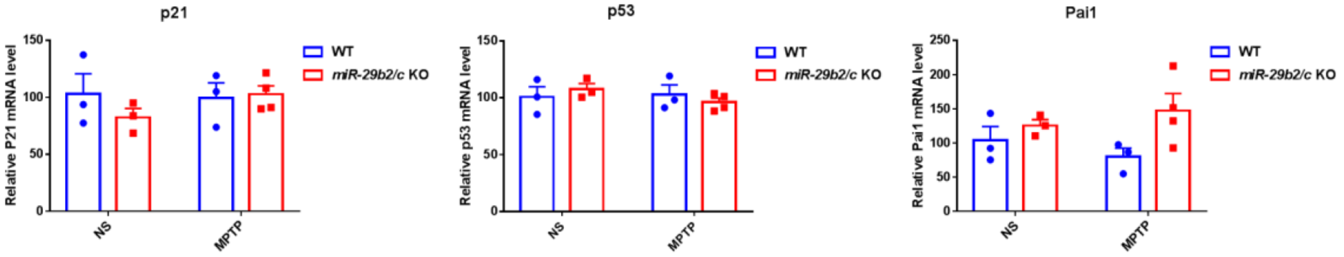

Supplementary Figure 6. The expression levels of senescence marker genes in the striatum of WT and *miR-29b2/c* KO mice at 3 days after MPTP injection. qPCR analysis of *p21*, *p53* and *Pai1* transcripts in the striatum of WT and *miR-29b2/c* KO mice. n=3-4.

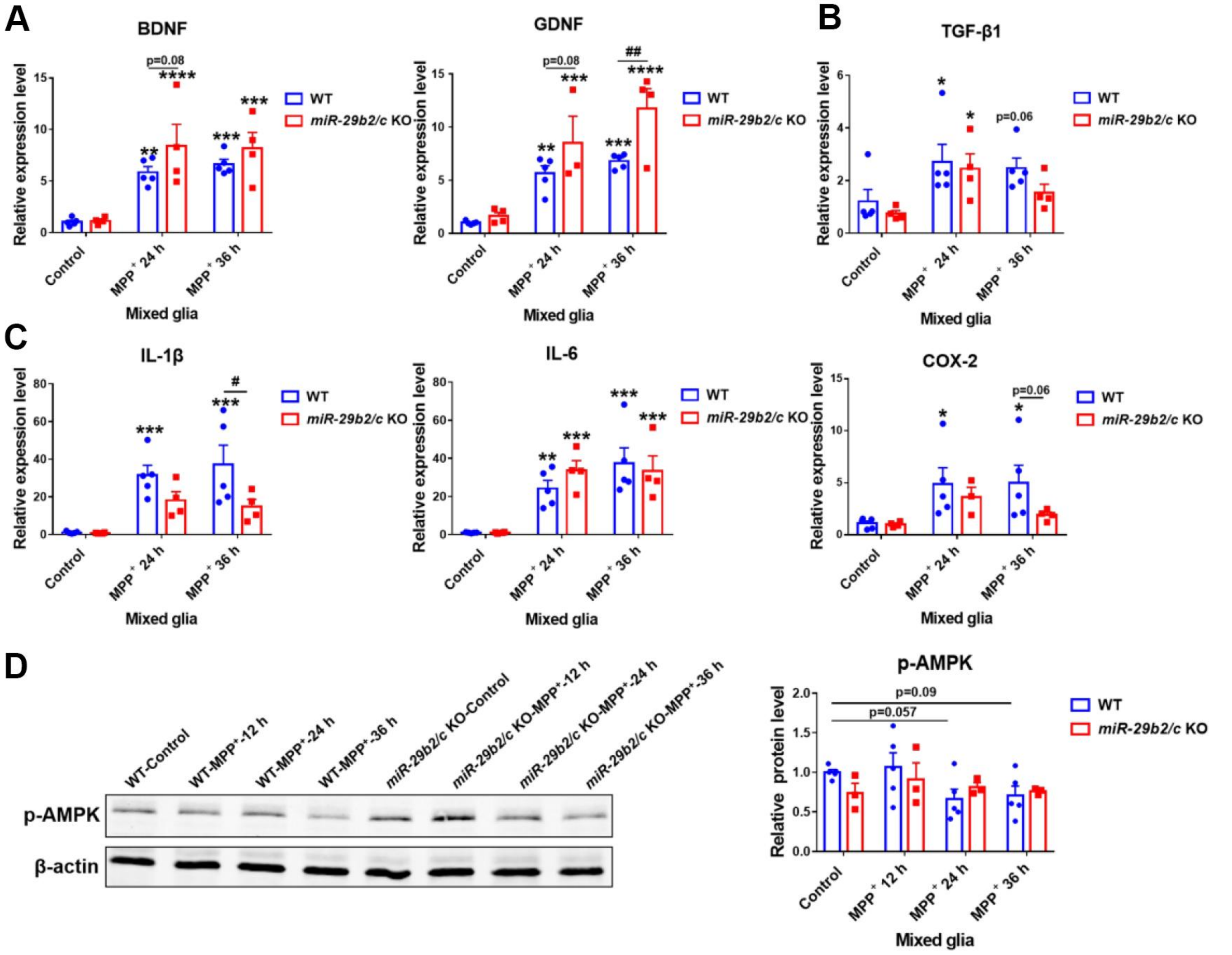

Supplementary Figure 7. Effects of *miR-29b2/c* deficiency in MPP+-treated primary mixed glia. qPCR analysis of neurotrophic factor *BDNF*, *GDNF* (**A**), anti-inflammatory factor *TGF-β1* (**B**) and pro-inflammatory factors *IL-1β*, *IL-6* and *COX-2* (**C**) transcripts in WT and *miR-29b2/c* KO primary mixed glia treated with PBS or MPP+ for 24 h and 36 h. n=3-5. (**D**) Western blot analysis of p-AMPK protein expression in WT and *miR-29b2/c* KO primary mixed glia treated with PBS or MPP+ for 12, 24 and 36 h. β-actin served as a loading control. Quantification of relative p-AMPK is shown in the right panel. n=3-5. The differences were analyzed by two-way ANOVA followed by LSD multiple comparison tests. *p<0.05, **p<0.01, ***p<0.001 and ***p<0.0001, *vs* PBS control. #p<0.05 and ##p<0.01, *vs* WT group.

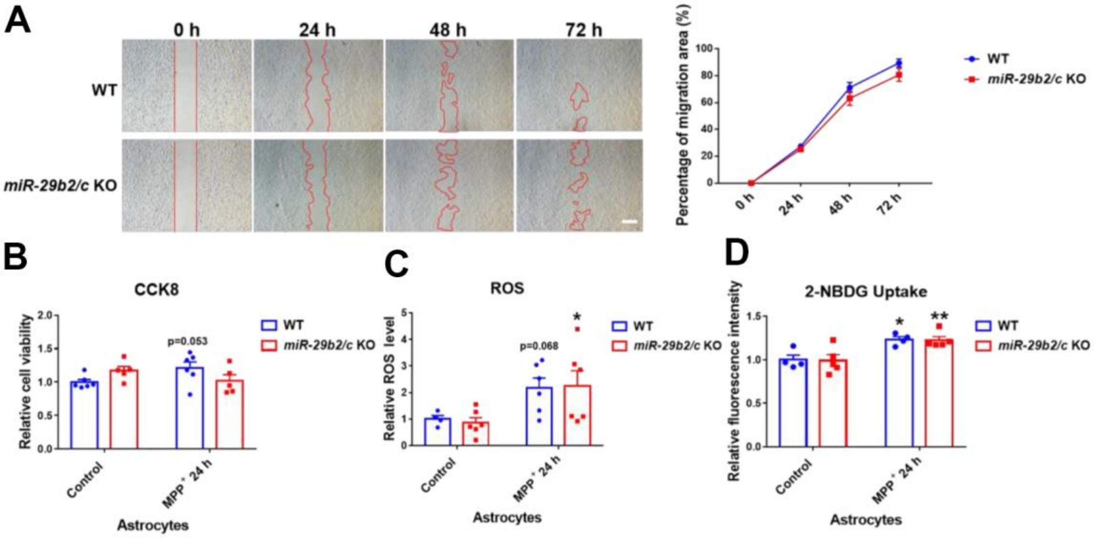

Supplementary Figure 8. (**A**) The scratch assay of WT and *miR-29b2/c* KO primary astrocytes at 0 h, 24 h, 48 h and 72 h. Scale bar: 100 µm. Percentage of scratch area is shown in the right panel. n=7-8. (**B**–**D**) Cell viability, ROS levels and glucose uptake capacities of WT and *miR-29b2/c* KO primary astrocytes. The results of CCK8 assay (**B**), ROS production levels (**C**) and 2-NBDG uptake levels (**D**) of WT and *miR-29b2/c* KO primary astrocytes treated with PBS or MPP+ for 24 h. n=4-6. The differences were analyzed by two-way ANOVA followed by LSD multiple comparison tests. **p* < 0.05, ***p* < 0.01, *vs* PBS control.

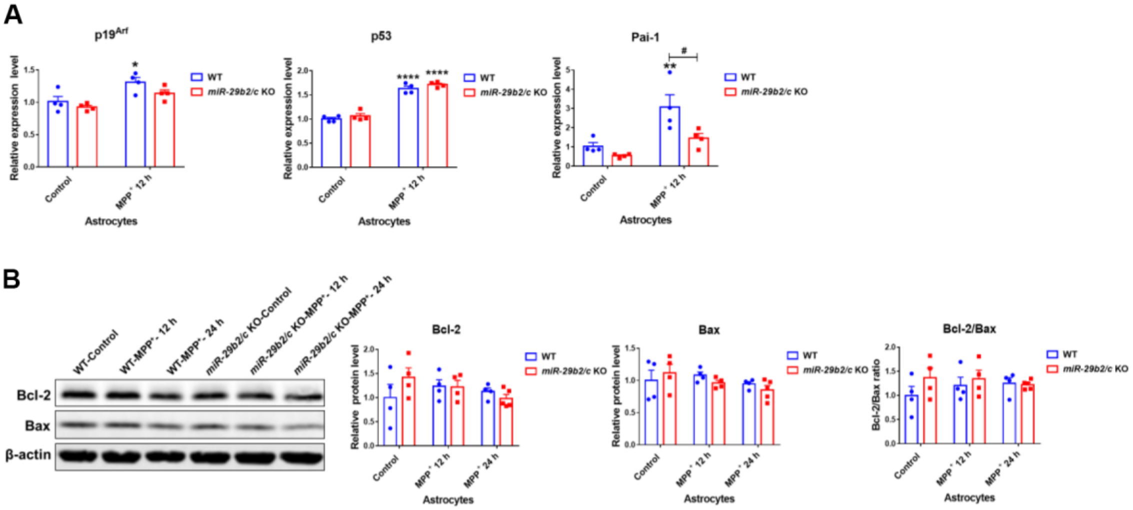

Supplementary Figure 9. (**A**) qPCR analysis of aging markers *p19^{Arf}, p53* and *Pai1* transcripts in WT and *miR-29b2/c* KO primary astrocytes treated with PBS or MPP+ for 12 h. n=4. The differences were analyzed by two-way ANOVA followed by LSD multiple comparison tests. **p*<0.05, ***p* < 0.01 and *****p*<0.0001, *vs* PBS control. # *p* < 0.05, *vs* WT group. (**B**) Western blot analysis of Bcl-2 and Bax protein expression in WT and *miR-29b2/c* KO primary astrocytes treated with PBS or MPP+ for 12 h and 24 h. β-actin served as a loading control. Quantification of relative Bcl-2 proteins and Bax proteins and their ratio are shown in the right panel. n=4-5.

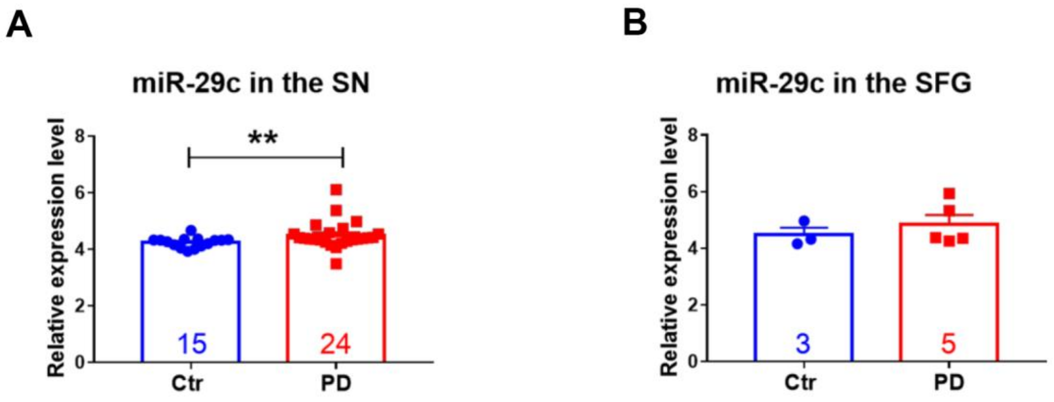

Supplementary Figure 10. The expression of miR-29c in the substantia nigra (SN), and the expression of miR-29c in the superior frontal gyrus (SFG) in PD patients and control subjects. (**A**) The expression of miR-29c in the SN. The differences were analyzed by Mann-Whitney test. ***p* < 0.01. (**B**) The expression of miR-29c in the SFG. Data are from GEO profiles [Parkinson's disease: substantia nigra (HG-U133B)].
